# Surgical Treatment in Active Infective Endocarditis: Results of a Four-Year Experience

**DOI:** 10.5402/2011/492543

**Published:** 2011-05-18

**Authors:** Carlo Rostagno, Enrico Carone, Alessandra Rossi, Gian Franco Gensini, Pier Luigi Stefano

**Affiliations:** ^1^Dipartimento Area Critica, Università di Firenze, 50134 Firenze, Italy; ^2^Dipartimento Cardiochirurgia, AOU Careggi, 50134 Firenze, Italy; ^3^Dipartimento Cardioanestesia, AOU Careggi, 50134 Firenze, Italy

## Abstract

*Background*. Aim of present investigation was to analyze survival and recurrence rate in patients with active endocarditis referred to our centre for surgical treatment. *Methods*. 80 consecutive patients with active infective endocarditis (52 males, 28 females, mean age 59.2 years) were referred to our institution for surgical treatment. 78 patients underwent surgery, and 2 patients died before intervention. *Results*. Fifty patients had native valve endocarditis, 30 prosthetic valve involvement. Hospital mortality has been 10.2%. Three discharged patients (4.9%) died at an average 18-month followup. Endocarditis recurred in 4 (2 being *S. aureus* prosthetic tricuspid endocarditis in drug addicts). All patients who underwent valve repair or homograft implant were alive and free of recurrence. *Conclusions*. Our results suggest that with proper surgical treatment patients with active endocarditis discharged alive from hospital have a survival >90% at 18 months with a low recurrence rate.

## 1. Introduction

Surgery is the treatment of choice in complicated prosthetic valves endocarditis [1, 2], and sterilization and “cure” by medical treatment alone are rarely possible in active infective endocarditis (IE) on native valves [3]. Residual valve damage may be associated with significant hemodynamic changes leading to a decreased life expectancy. Surgical treatment has been demonstrated to be the only independent predictor of long-term survival in a large study conducted in France on patients admitted to hospital for IE [4]. Recently a large multicentric study showed that surgery was associated with a decreased risk of both in-hospital and late mortality [5].

In the active phase of illness guidelines suggest an early surgical approach, associated with and followed by antibiotic therapy for 6–8 weeks after surgery [6]. The purpose of our study was to assess hospital mortality, recurrence rate of endocarditis, and midterm survival (average followup 18 months) in patients consecutively referred in a period of 4 years to Florence Heart Surgery Department for surgical treatment of active endocarditis.

## 2. Material and Methods

In the study were included 80 consecutive patients referred to our institution between January 1, 2003 and December 31, 2006 for surgical treatment of active infective endocarditis [7]. Mean age was 59,5 years (range 22–84 years). There was a male prevalence (50/30), and men were on average 6 years younger than women (57.5 versus 63.1 years) (Table 1). Sixty-eight percent were referred by primary or second level hospitals of Tuscany, while the other 32% from the emergency department of our hospital. Fifty patients suffered from native valve endocarditis—NVE—(62%) while 30 were affected by prosthetic valve endocarditis—PVE—(38%). Early PVE, defined as an infection occurring within 1 year after valve replacement, accounted for 40% of PVE. Late prosthetic valve endocarditis occurred in 18 patients. 

At admission in our department patients underwent accurate history collection; physical examination with particular attention to heart, neurological findings, skin or ocular manifestations, history or clinical evidence of systemic embolism; assessment of vital signs, measurement of body temperature, ECG, and laboratory examinations (haemoglobin concentration, leukocyte count, serum creatinine). At least 3 samples for blood cultures were collected within 12 hours. Finally transthoracic and transesophageal echocardiographies were performed.

In patients with positive blood cultures, antibiotic treatment was administered according to results of antibiotic susceptibility testing. Large spectrum empiric antibiotic treatment was prescribed in patients with culture negative endocarditis [6, 8]. Eight patients with methicillin-resistant *Staphylococcus aureus* (MRSA) infection were treated with linezolid, vancomicin, or teicoplanin associated with an aminoglycoside. 

Surgery was scheduled within seven days from admission. 32 patients with NVE underwent valve replacement while in 18 (36%) a conservative surgery with valve repair had been possible (Table  2(a)). Valve repair was more frequently performed in mitral valve endocarditis (13/20 patients) than in aortic (2/10) or combined mitral and aortic valve disease. Homograft replacement of aortic valve and aortic root has been employed in 12/13 patients with complicated aortic PVE.

Additional operative procedures included the closure of infection-related ventricular septal defects in 2 patients and closure of a congenital atrial septal defect in 1 patient. In one patient with aortic valve endocarditis coronary revascularization with venous graft on right coronary artery was performed.

After discharge, all patients were admitted to a rehabilitation centre and thereafter followed by their general practitioner. Clinical examinations were scheduled (3, 9, 18, 24 months). The mean follow-up period after hospital discharge has been 18 months (range 3 to 48 months). In 26 patients not followed at our centre, information on clinical conditions was obtained through a telephonic questionnaire in which data about survival, hospital readmissions, recurrences of endocarditis, and exercise capacity at the time of interview were collected. All patients gave informed consent to the study at hospital admission, and the study was approved by the ethical committee of the hospital. 

### 2.1. Statistical Analysis

The quantitative variables are reported as means and standard deviations. In case of not continuous parameters the frequency of distribution has been reported.

The statistical analysis of clinical data was carried out by the Student's *t*-test for continuous data, while for not continuous variables *χ* ^2^ test or Fisher exact test when appropriated was used.

The analysis of survival was made using the Kaplan-Meier method, and the difference between the groups was analyzed using the log-rank test. A probability value of <.05 was considered statistically significant.

## 3. Results

### 3.1. Clinical Characteristics

At hospital admission 56% of patients was in NYHA functional class III-IV, 30% and 14% respectively, in class I and in class II.

Predisposing factors, a part from previous valve surgery, were rarely identified. Three patients had history of dental procedures and *Streptococcus viridans* endocarditis. Two S. aureus tricuspid valve endocarditis were observed in intravenous drug addicts. One patient with endocarditis due to *S. bovis* was affected by chronic inflammatory bowel disease. For most of late prosthetic valve endocarditis, we were not able to identify a causal event for the development of infection. Overall incidence of peripheral embolism confirmed by instrumental investigations was 35%.

### 3.2. Etiologic Infective Organisms

Blood cultures allowed identification of the etiologic agent in 58/80 patients. Staphylococci were the most common microorganisms isolated in blood cultures (Table 3). Streptococci were identified in 26 patients while a Gram-negative was found in 5 patients. One immune depressed patient had fungal endocarditis (*Candida parapsilosis*). Blood cultures were negative in 22 subjects, 15 with native valve disease. Almost all these patients were referred from peripheral hospitals and previously treated with large spectrum empiric antibiotic therapy.

### 3.3. Echocardiographic Characteristics

Echocardiography performed at admission showed a preserved left ventricular systolic function (EF > 50%) in 65% of patients. In 31% left ventricular function was moderately depressed (EF between 35 and 50%). Only 5 patients showed a severe functional impairment (EF < 35%).

An annular abscess was identified at TEE in 12/19 patients with aortic PVE and only in 1/19 patient with aortic NVE. One or more mobile vegetations were identified in 90% of NVE. Overall moderate to severe valvular regurgitation occurred in 81% of patients with mitral valve involvement and 79% of those with aortic valve endocarditis. More than trivial tricuspidal regurgitation could be observed in 55% of patients.

### 3.4. Indications for Surgery

Hemodynamic impairment related to severe mitral or aortic valve regurgitation was the main indication for surgery in 48% of patients. In 15 patients surgery was indicated for perivalvular spreading of the infection with abscesses (14 subjects, 92% in PVE) or aortic to right atrium fistula (1 patient). Large vegetations >10 mm diameter (24%) and recurrent embolism were the other main indications for surgery. All patients underwent surgery within 7 days after starting antibiotic treatment, unless impairment of clinical conditions suggested an earlier solution.

### 3.5. Hospital Mortality and Morbidity

Of the 80 patients included in the study two died before surgery, one due to septic shock and widespread peripheral embolism, the other suddenly from the rupture of an aortic abscess. In-hospital postsurgery mortality has been 10.2% (8/78). One patient died in operating room (mitral valve replacement) for rupture of the atrioventricular sulcum. The other 7 patients died within the first 30 days after surgery, 3 due to irreversible cardiogenic shock, 3 due to multisystem organ failure as a result of severe septic shock, and the last one for rupture of a mycotic aneurism of splenic artery.

Factors closely related to in-hospital mortality were advanced age, severe depression of left ventricular function, and finally clinical and laboratory evidence of severe sepsis (temperature at admission over 38°C, WBC > 15000 mm^3^, serum creatinine > 2,0 mg/dL) (Table 4). We did not find any significant difference in hospital survival between native or PVE, between mitral or aortic valve involvement or finally between patients with or without perivalvular involvement.

**Figure 1:**
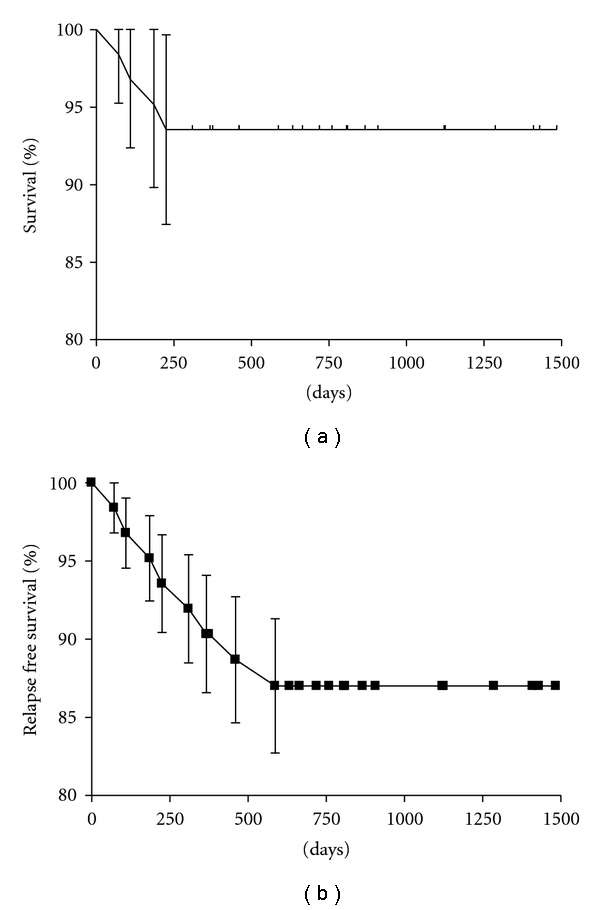
Overall survival (a) and event free-death, endocarditis recurrence-survival (b) in patients discharged alive from hospital.

**Figure 2:**
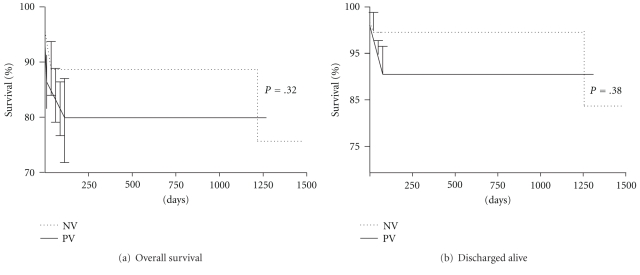
Comparison of overall survival (a) and survival in patients discharged alive (b) between patients with NVE and PVE.

 In patients discharged alive from hospital survival was 95.1% (67 out of 70) at an average followup of 18 months (Figure 1(a)). Three patients died during followup, one for lung cancer the other two from refractory heart failure. Event-free survival (death and endocarditis recurrence) is reported in Figure 1(b). Endocarditis recurred only in 4 patients: all needed reintervention. Two were intravenous drug users with recurrence of staphylococci endocarditis on prosthetic tricuspid valve. In the other 2 biologic aortic prosthetic valve showed severe paravalvular leak, respectively, 3 and 5 months after surgery. Blood cultures were positive for S. aureus in both patients. We did not find any significant difference in survival between native or prosthetic valve endocarditis (Figures 2(a) and 2(b)).

Survival was not significantly different between patients undergoing valve repair and those undergoing valve replacement. In the group of patients selected for valve repair strategy none had recurrence of endocarditis, and at followup echocardiography did not show more than mild residual regurgitation. Similarly none of patients treated with homograft implant had postoperative complications.

Surgical treatment led to a significant functional improvement. At the end of followup 90% of patients discharged alive from hospital were in NYHA class I-II in comparison to 58% in the preoperative period. Only 10% had still a severe functional limitation after surgery (NYHA class III-IV).

## 4. Discussion

Previous clinical investigations suggested that surgical treatment in active infective endocarditis is associated with a survival rate ranging from 70 to 85% and with a recurrence rate between 8.5 and 15% [9–11]. The availability of different surgical solutions (e.g., valve repair or aortic homografts) other than valve replacement, allowing the choice of the proper technical strategy in the single patient, might improve results of surgical treatment of active infective endocarditis, in particular decreasing the risk of infectious recurrences and the need for long-term anticoagulation. 

Early surgery should be considered the treatment of choice in active complicated PVE [12] in patients that are not too sick to represent a prohibitive surgical challenge. Previous investigations suggested that homograft aortic root replacement may improve survival rate and in particular decrease the risk of recurrences [13]. In the study by Lopes et al. [14] 41 patients with complex PVE were treated with allograft aortic root replacement. In-hospital mortality has been 4.8%; two patents needed late reintervention for graft failure. No patients showed recurrence of endocarditis, and overall survival at ten years was 79%. The role of homograft in treatment of aortic endocarditis has been questioned by Avierinos et al. [15]. The authors did not find significant difference in survival rate in relation between conventional treatment with valve replacement and the use of homograft. However the study suffered from several methodological limitations; mainly patients were not randomized, and most of patients with complicated PVE were treated with homograft implantation (annular complications were found in 76% of the homograft group versus 30% in conventionally treated group). The absence of differences in long term outcome suggests that homografts may be a-safer therapeutic option in patients with more severe and advanced disease although some concerns still regard their durability. In present investigation 13/14 (92%) patients with complicated aortic endocarditis (12 PVE, 1 NVE) were treated with aortic homograft. At followup none of these patients died or showed recurrence.

Valve repair, in particular in patients with mitral valve endocarditis, is considered a valuable therapeutic option when technically feasible. Conservative surgery allows to decrease the risks related to prolonged anticoagulation and the unfavourable left ventricular geometric changes associated with valve replacement [16]. In our study in 65% (13/20) patients with isolated mitral NVE has been possible to preserve the valve. Moreover in 2 patients with tricuspid, 2 with aortic, and 1 with combined aortic and mitral valve involvement valve repair was preferred to valve replacement. None of the patients undergoing NVE reparative surgery had significant postoperative complications. Only one patient undergoing mitral valve repair had mild regurgitation at 64 week followup. 

Overall mortality in patients discharged alive from hospital has been less than 5%, at an average followup of 18 months. In particular patients undergoing valve repair and homograft implant were all alive and had no recurrent endocarditis. The severity of the septic state at hospital admission is an important prognostic factor for perioperative mortality with a more relevant clinical impact than the degree of hemodynamic impairment.

### 4.1. Study Limitations

Present investigation is a relatively small single centre observational study of patients undergoing surgery for active infective endocarditis. The length of followup is limited (average 18 months), and we are aware that homograft degeneration, often leading to the need of reintervention, may be observed during longer follow-up periods. However homograft-treated patients was a group with complicated endocarditis at high risk of death or recurrence after conventional treatment with valve replacement. In our series at followup patients treated with homograft were all alive and free from infective recurrences.

 Seventy-five percent of patients included in present investigation were referred for surgery to our centre from peripheral hospitals. Preselection of patients may have contributed to the invoice for surgery of patients with more severe disease but on average younger, with a lower number of comorbidities and at overall lower surgical risk in comparison to the whole population of patients suffering from endocarditis. This hypothesis is in agreement with the observations of the International Collaboration in Endocarditis Prospective Cohort database [5]. The availability of several surgical solutions (e.g., valve repair or aortic homografts) other than valve replacement, allowing the choice of the proper technical strategy in the single patient, is associated with an acceptable operative risk and with significant improvement in midterm survival and decreased risk of recurrences.

## Figures and Tables

**Table 1:** Clinical characteristics of patients included in the study.

			Mean age (±SD)
*Overall*	no	80	59.5 ± 16.1
*Men*	no	50	57.3 ± 15.8
*Women*	no	30	63.1 ± 16.8

*Type of valve involvement*			no
Native valve endocarditis			50
Prosthetic valve endocarditis			30

*Valve involved*			no
Mitral			28
Aorta			38
Tricuspid			4
Mitral + aorta			10

*NYHA Class*			no
IV			21
III			23
II			14
I			22

**Table:** (a)

Surgical procedure	NVE (Tot = 50, Ao = 20, Mt = 20, Tc = 4, Mt + Ao = 6)	PVE (Tot = 30, Ao = 18, Mt = 12)
Aortic valve replacement	18	7
Mitral valve replacement	7	10
Mitral and aortic valve replacement	5	1
Tricuspid valve replacement	2	—
Mechanical prosthesis	24	10
Biologic prosthesis	8	8
Homograft	1	12
Valve repair	18	0

**Table:** (b)

	NVE (20 Ao/20 Mt/4 Tc/6 Ao + Mt)	PVE (18 Ao/12 Mt)
Haemodynamic instability with or without cardiogenic shock	26	10
Perivalvular abscess	1	13
Previous embolization	8	3
Vegetations > 10 mm	15	4

**Table 3:** Microorganism responsible for endocarditis in relation to the type of valve affected.

Aetiologic agent	NVE (no 50)		PVE (no 30)		Total (no 80)	
	no	%	no	%	no	%
Staph. aureus	9	18	7	23	16	20
Staph. Epidermidis	3	6	5	17	8	10
Other Staph	2	4	0	0	2	2.5
Strept. faecalis	2	4	5	17	7	8.7
Strept. viridans	4	8	0	0	4	5
Other Strept.	12	24	3	10	15	18.8
Gram-negative	2	4	3	10	5	6.2
Candida	1	2	0	0	1	1.2
Negative blood Colture	15	30	7	23	22	27.6

**Table 4:** In-hospital outcome in relation to demographic, hemodynamic, echocardiographic, and clinical and laboratory parameters.

Parameter	Patients discharged alive	Patients died after surgery during hospitalization	*t*	*P*
	(70)	(8)		
Age (years)	58.1 ± 16.3	69.7 ± 10	2.194	.03
Heart rate (bpm)	87 ± 19	105 ± 20	2.67	.009
Systolic heart pressure (mmHg)	127 ± 22	130 ± 23	0.384	.710
Diastolic heart pressure (mmHg)	67 ±16	71 ± 16	0.69	.492

LV Ejection Fraction (%)			*X* ^2^	
>50%	57	1	26.6	<.0001
35–50%	12	4		
<35%	1	3		

Aortic regurgitation			*X* ^2^	
3+-4+	31	2	1,37	.50
<2+	22	4		
0	17	2		

Mitral regurgitation			*X* ^2^	
3+-4+	30	3	0.08	.95
<2+	24	3		
0	16	2		

Temperature °C			*X* ^2^	
>38	12	5	9,93	.007
37-38	31	3		
<37	27	0		

Total WBC/mm3			*X* ^2^	
>15,000	6	5	21.4	<.001
10,000–15,000	15	3		
<10,000	49	0		

Hemoglobin g/dl			*X* ^2^	
<10	22	6	6.8	.04
10–12	22	2		
>12	26	0		

Creatinine mg/dl			*X* ^2^	
>2	7	1	20.33	<.001
1.5–2	5	5		
<1.5	58	2		

C-reactive protein (mg/l)			Fisher ex	n.s.
>10	23	3	text	
<10	47	5	1.0	
